# Induction of unique macrophage subset by simultaneous stimulation with LPS and IL-4

**DOI:** 10.3389/fimmu.2023.1111729

**Published:** 2023-04-21

**Authors:** Kei Ishida, Takahiro Nagatake, Azusa Saika, Soichiro Kawai, Eri Node, Koji Hosomi, Jun Kunisawa

**Affiliations:** ^1^ Laboratory of Vaccine Materials and Laboratory of Gut Environmental Health, Microbial Research Center for Health and Medicine, National Institutes of Biomedical Innovation, Health and Nutrition (NIBIOHN), Ibaraki, Osaka, Japan; ^2^ Graduate School of Pharmaceutical Sciences, Osaka University, Suita, Osaka, Japan; ^3^ Laboratory of Functional Anatomy, Department of Life Sciences, School of Agriculture, Meiji University, Kawasaki, Kanagawa, Japan; ^4^ Graduate School of Medicine, Osaka University, Suita, Osaka, Japan; ^5^ Graduate School of Dentistry, Osaka University, Suita, Osaka, Japan; ^6^ Graduate School of Science, Osaka University, Toyonaka, Osaka, Japan; ^7^ International Vaccine Design Center, Institute of Medical Science, The University of Tokyo, Minato, Tokyo, Japan; ^8^ Department of Microbiology and Immunology, Graduate School of Medicine, Kobe University, Kobe, Hyogo, Japan; ^9^ Research Organization for Nano and Life Innovation, Waseda University, Shinjuku, Tokyo, Japan; ^10^ Graduate School of Biomedical and Health Sciences, Hiroshima University, Hiroshima City, Hiroshima, Japan

**Keywords:** macrophages, energy metabolism, LPS and IL-4, M1 macrophage, M2 macrophage

## Abstract

Macrophages manifest as various subtypes that play diverse and important roles in immunosurveillance and the maintenance of immunological homeostasis in various tissues. Many *in vitro* studies divide macrophages into two broad groups: M1 macrophages induced by lipopolysaccharide (LPS), and M2 macrophages induced by interleukin 4 (IL-4). However, considering the complex and diverse microenvironment *in vivo*, the concept of M1 and M2 is not enough to explain diversity of macrophages. In this study, we analyzed the functions of macrophages induced by simultaneous stimulation with LPS and IL-4 (termed LPS/IL-4-induced macrophages). LPS/IL-4-induced macrophages were a homogeneous population showing a mixture of the characteristics of M1 and M2 macrophages. In LPS/IL-4-induced macrophages, expression of cell-surface M1 markers (I-A^b^) was higher than in M1 macrophages, but lower expression of iNOS, and expression of M1-associated genes (*Tnfα* and *Il12p40*) were decreased in comparison to expression in M1 macrophages. Conversely, expression of the cell-surface M2 marker CD206 was lower on LPS/IL-4-induced macrophages than on M2 macrophages and expression of M2-associated genes (*Arg1*, *Chi3l3*, and *Fizz1*) varied, with *Arg1* being greater than, *Fizz1* being lower than, and *Chi3l3* being comparable to that in M2 macrophages. Glycolysis-dependent phagocytic activity of LPS/IL-4-induced macrophages was strongly enhanced as was that of M1 macrophages; however, the energy metabolism of LPS/IL-4-induced macrophages, such as activation state of glycolytic and oxidative phosphorylation, was quite different from that of M1 or M2 macrophages. These results indicate that the macrophages induced by LPS and IL-4 had unique properties.

## 1 Introduction

Macrophages have been extensively studied since the middle of the 19th century when it was discovered that certain types of leukocytes are able to actively capture foreign particles and senescent erythrocytes and are a part of a general defense system against pathogens (1, 2). Recent studies on macrophages have revealed that macrophages do not exhibit a uniform phenotype but change their properties depending on the surrounding microenvironment (3). When studying the functional differences of macrophages *in vitro*, macrophages are generally classified into inflammatory-type M1 macrophages and anti-inflammatory-type M2 macrophages (3).

M1 macrophages are generally induced by lipopolysaccharide (LPS) and are characterized by high expression of MHC class II molecules, which are involved in antigen presentation, and CD86 molecules, which are involved in co-stimulation of T cells. M1 macrophages also produce inflammatory cytokines such as tumor necrosis factor alpha (TNF-α), interleukin 6 (IL-6), and IL-12 (3). They also produce nitric oxide (NO) from arginine, accompanied by high expression of inducible nitric oxide synthase (iNOS) and reactive oxygen species (ROS). These properties allow macrophages to eliminate pathogens such as bacteria and viruses in their immunosurveillance role. M1 macrophages are also involved in the development of inflammatory diseases such as diabetes, cancer, and atherosclerosis (3).

M2 macrophages, on the other hand, are induced by IL-4 and are characterized by high expression of CD206, a mannose receptor. They highly express arginase (Arg1) and inhibit NO production by competing with iNOS in arginine metabolism. They are characterized by high expression of chitinase 3-like 3 (*Chi3l3*) (also called *Ym1*) and *Fizz1* (also called *RELMα*) (3), which are involved in suppression of various inflammatory and allergic responses and in tissue repair (3).

Recently, energy metabolism has been recognized as an important factor controlling the function of immune cells, and this emerging field of immunometabolism is attracting a great deal of attention (4). For example, the dependence of immune cells on energy metabolism pathways such as glycolysis, the citric acid cycle (TCA cycle), and oxidative phosphorylation varies depending on the immune cells’ state of activation and differentiation (4). In M1 macrophages, the predominant pathway is glycolysis, which is required for enhancing phagocytic activity and for promoting ROS production by nicotinamide adenine dinucleotide phosphate (NADPH) oxidase (NOX) (5–7). In M2 macrophages, on the other hand, the predominant pathways are β-oxidation, the TCA cycle, and oxidative phosphorylation, which are thought to be involved in long-term inflammatory convergence, cell proliferation, and tissue repair by continuous massive energy production (5–7).

The concept of M1 and M2 macrophages is often used as an indicator of the different properties of macrophages (3). However, the complex and diverse microenvironment surrounding macrophages is diverse, for example, bacterial infection in severe asthma, where macrophages are simultaneously exposed to LPS and IL-4 (8, 9). Therefore, there should be heterogeneous macrophage populations *in vivo*, but this issue is not fully understood (10–12). In this study, we aimed to characterize the immunological properties of macrophages simultaneously stimulated with LPS and IL-4.

## 2 Methods

### 2.1 Mice

Male C57BL/6J wild-type mice (5 weeks old) were purchased from Japan SLC (Hamamatsu, Japan) and were allowed to acclimate at the specific-pathogen-free animal facility at the National Institutes of Biomedical Innovation, Health and Nutrition (NIBIOHN, Osaka, Japan). Mice were kept under stable conditions (16:8-h light/dark cycle, 22–24°C and 50%-60% humidity) with free access to food and distilled water. Mice were killed by cervical dislocation under isoflurane (Forane; AbbVie, North Chicago, IL, USA) anesthesia. All experiments were conducted in accordance with the guidelines of the Animal Care and Use Committee of NIBIOHN.

### 2.2 Preparation of bone marrow–derived macrophages, and induction of M1 and M2 macrophages

Bone marrow–derived macrophages were prepared as described previously (13) with modification. Briefly, C57BL/6J wild-type mice (6-12 weeks old) were killed, and bone marrow cells were extracted from the femurs and tibias. These cells were treated with cell lysis buffer (0.16 mol/L NH_4_Cl and 0.17 mol/L Tris (hydroxymethyl) aminomethane (Nacalai Tesque, Kyoto, Japan) for 1 min at room temperature. After that, these cells were cultured in 6-cm dishes (1×10^5^ cells/mL, 5 mL/dish; RepCell dishes; CellSeed, Tokyo, Japan) with Dulbecco’s modified Eagle’s medium (high glucose) supplemented with macrophage colony-stimulating factor (50 ng/mL; PeproTech, Cranbury, NJ, USA), 10% (vol/vol) fetal bovine serum (Corning, Corning, NY, USA), and 1% (vol/vol) penicillin–streptomycin (Nacalai Tesque, Kyoto, Japan) at 37°C in 5% CO_2_ (Day 0). Culture medium was replaced on Days 3 and 5. On Day 6, cells were stimulated with LPS (20 ng/mL, O127:B8; Sigma Aldrich, Darmstadt, Germany) or IL-4 (20 ng/mL; PeproTech) for 24 h to induce M1 and M2 macrophages, respectively. As an experimental group, cells were stimulated with both LPS (20 ng/mL) and IL-4 (20 ng/mL) at the same time.

### 2.3 Cell isolation and flow cytometric analysis

Cell isolation and flow cytometry were performed as described previously (13). To avoid non-specific staining, cell samples were blocked with anti-CD16/32 monoclonal antibody (mAb) (dilution 1:100; catalog no. 101320, TruStain fcX; BioLegend, San Diego, CA, USA). The following fluorescently labeled mAbs were used for flow cytometric analysis: fluorescein 5(6)-isothiocyanate (FITC) -anti-CD206 (1:20; 141704, BioLegend), Phycoerythrin (PE) -anti-I-A^b^ (1:100; 116408, BioLegend), Allophycocyanin (APC) -Cyanine (Cy) 7-anti-CD11b (1:100; 101226, BioLegend), PE-Cy7-anti-F4/80 (1:100; 123114, BioLegend), and Brilliant Violet (BV) 421-anti-CD45 (1:100; 103133, BioLegend). Dead cells were detected by using 7-AAD (1:100; 420404, BioLegend) and excluded from analysis. Flow cytometric analysis was conducted by using FACSAria (BD Biosciences, Bristol, UK). Data were analyzed by using FlowJo 9.9 (Tree Star, Ashland, OR, USA).

### 2.4 Reverse transcription and quantitative PCR analysis

After performing reverse transcription, quantitative PCR analysis was performed both LightCycler 480 II (Roche, Basel, Switzerland) with FastStart Essential DNA Probes Master (Roche) as previously described (13, 14) and CFX Opus Real-Time PCR Systems (Bio-Rad, Hercules, California, USA) with PrimePCR™ Probe Assay. Primer sequences for LightCycler 480 II were as follows: *Arg1* sense, 5′-gaatctgcatgggcaacc-3′; *Arg1* antisense, 5′-gaatcctggtacatctgggaac-3′; *Chi3l3* sense, 5′-aagaacactgagctaaaaactctcct-3′; *Chi3l3* antisense, 5′-gagaccatggcactgaacg-3′; *Fizz1* sense, 5′-ccctccactgtaacgaagactc-3′; *Fizz1* antisense, 5′-cacacccagtagcagtcatcc-3′; *Il12p40* sense, 5′-ttgctggtgtctccactcat-3′; *Il12p40* antisense, 5′-gggagtccagtccacctcta-3′; *Tnfα* sense, 5′-ctgtagcccacgtcgtagc-3′; *Tnfα* antisense, 5′-ttgagatccatgccgttg-3′; *Actb* sense, 5′-aaggccaaccgtgaaaagat-3′*; Actb* antisense, 5′-gtggtacgaccagaggcatac-3′. Unique assay ID of PrimePCR Probe (BioRad) for CFX Opus Real-Time PCR Systems were as follows: *STAT3*: qDreCIP0044562, *Socs1*: qMmuCEP0057945.

### 2.5 Flux analysis

Real-time analysis of energy metabolism was performed by using a flux analyzer (Seahorse Bioscience XF24 Extracellular Flux Analyzer; Agilent Technologies, Santa Clara, CA, USA) and XF Mito Stress Kit (Agilent Technologies) according to the manufacturers’ protocols. Briefly, the cell culture microplate (Agilent Technologies) was treated with 16.5 μg/mL Cell-TAK (Corning) in 0.1 mol/L NaOH and incubated at room temperature for 25 min. The plate was washed with distilled water after removing Cell-TAK liquid, and cells were cultured at 8×10^4^ cells/well with XF RPMI Medium (Agilent Technologies) containing glutamine (2 mmol/L, Agilent Technologies), glucose (10 mmol/L, Agilent Technologies), and pyruvate (1 mmol/L, Agilent Technologies) until starting flux analysis. The plate (Agilent Technologies) was set into the flux analyzer, and the following compounds (final concentrations) were injected during the assay: 1.5 µM oligomycin (inhibitor of ATP synthase), 1.5 µM FCCP (proton uncoupling agent), and 0.5 µM rotenone + 0.5 µM antimycin A (inhibitors of the mitochondrial respiration complex). XFe Wave software (Agilent Technologies) was used to analyze the results.

### 2.6 Phagocytosis assay

Cells were seeded into 24-well plates (Corning) at 1×10^5^ cells/well and centrifuged (200*g*, 10 min, room temperature) for adhesion, followed by incubation in Dulbecco’s modified Eagle’s medium (high glucose) containing 10% (vol/vol) fetal bovine serum and 1% (vol/vol) penicillin–streptomycin at 37°C under 5% CO_2_ overnight.*Escherichia coli* (Competent Quick DH5α; Toyobo, Osaka, Japan) were grown in LB medium overnight at 37°C with shaking at 200 rpm. They were incubated with 5.0 mg/mL FITC (Sigma Aldrich) for 1 h at 37°C in a water bath and protected from light, and were then washed twice with PBS. Cells were incubated with the FITC-staining *E. coli* at cell/bacteria ratio of 1:500 for 30 min after centrifuging (200*g*, 10 min, room temperature) for adhesion. Non-phagocytosed bacteria were removed by washing three times with PBS, and the cells were harvested by treating with 0.5 g/L trypsin–0.53 mmol/L EDTA (Nacalai Tesque) for 5 to 10 min at 37°C in 5% CO_2_. Phagocytosis activity was assessed by calculating the mean fluorescent intensity (MFI) from flow cytometric analysis; phagocytosis activity against fluorescently labeled microbeads (YG Carboxylate Microspheres, 1.00 μm; Polysciences, Warrington, PA, USA) was assessed in the same way. Trypan blue solution (Nacalai Tesque) was added to quench fluorescence of surface-bound FITC-staining *E. coli*. To inhibit glycolysis, cells were treated with 2-deoxy-D-glucose (50 mmol/L, Sigma Aldrich) for 30 min before adding FITC-staining *E. coli*.

### 2.7 Western blot analysis

Cells were lysed by using RIPA Lysis buffer (Sigma Aldrich) containing Protease Inhibitor Cocktail (1 mL/10 mL lysates, Sigma Aldrich) and PhosSTOP™ (1 tablet per 10 mL lysate, Roche). The lysates were incubated for 20 min at 4°C with shaking, followed by centrifuged (130000*g*, 10 min, 4°C) and the supernatant was used for automated capillary-based immunoassay on a Wes Simple Western System (ProteinSimple, Bio-Techne, Minneapolis, USA). Primary antibodies were used as followed Actin-β (dilution 1:100; catalog no. A5441, Sigma Aldrich), p65 (1:50; 8242, Cell Signaling Technology, Danvers, MA, USA), phosphorylated-p65 (p-p65) (1:50; 3033, Cell Signaling Technology), STAT6 (1:50; 5397, Cell Signaling Technology), p-STAT6 (1:50; 56554, Cell Signaling Technology), STAT1 (1:50; 9172, Cell Signaling Technology) and p-STAT1 (1:50; 7649, Cell Signaling Technology). These antibodies were diluted with antibody diluent 2 (ProteinSimple). Secondary antibody was horseradish peroxidase (HRP)-conjugated anti-rabbit IgG antibody (ProteinSimple) or anti-mouse IgG antibody (ProteinSimple) and Lumino-S/peroxidase (ProteinSimple) was used as substrates for HRP.

### 2.8 Photographs of each macrophage

Images of each cell were obtained with an all-in-one fluorescence microscope (BZ-X800, KEYENCE, Osaka, Japan).

### 2.9 Statistical analysis

Statistical significance was evaluated by using Prism 3.03 software (GraphPad Software, San Diego, CA). Data are expressed as mean ± standard error. Unpaired t-test was used for comparison between two groups and the Tukey method was used for comparison between multiple groups, with significance levels of *P<0.05, **P<0.01, and ***P<0.001.

## 3 Results

### 3.1 Cell-surface molecule expression and gene expression profiles in LPS/IL-4-induced macrophages show a unique pattern

**Figure 1:**
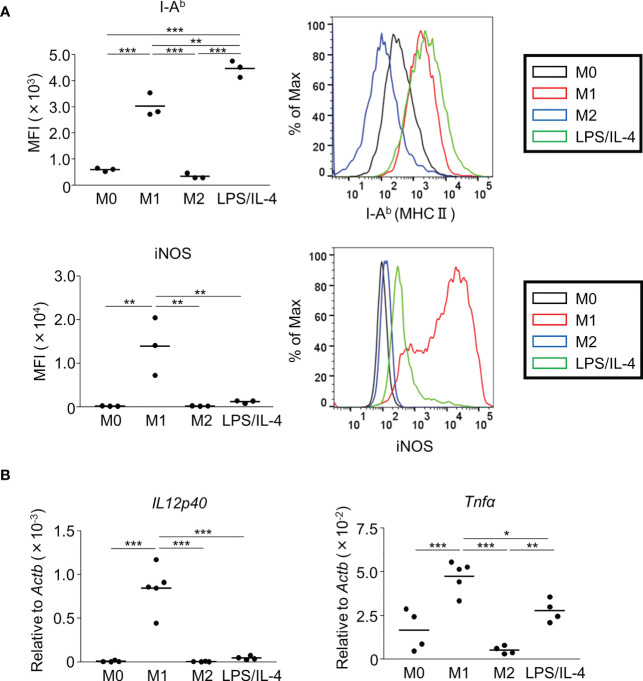
M1 marker expression profiles of M1 macrophages and LPS/IL-4-induced macrophages. Bone marrow-derived macrophages were prepared and stimulated with either LPS, IL-4, or LPS plus IL-4 for 24 h **(A, B)**. **(A)** Flow cytometric analysis was performed to examine the expression of M1 markers (i.e., I-A^b^ and iNOS). Mean fluorescence intensity (MFI) and representative histogram data are shown and gated on 7-AAD^−^ CD45^+^ CD11b^+^ F4/80^+^ for I-A^b^ and gated on only 7-AAD^−^ for iNOS. **(B)** Gene expression levels were examined by reverse transcription-quantitative PCR analysis. The expression levels of *Il12p40* and *Tnfα* were normalized to that of *Actb*. Each point represents data from an individual experiment. Horizontal bars indicate mean value. *P<0.05, **P<0.01, ***P<0.001.

We prepared non-activated (M0), M1, M2, and LPS/IL-4-induced macrophages and evaluated their morphology and M1 and M2 markers in each by cell-surface molecule expression and gene expression. In comparison to M0 macrophages, M1 macrophages showed round shape, while M2 macrophages showed cellular elongation. We found that LPS/IL-4-induced macrophages resemble to M1 macrophages (**Figure S1**). In addition, as previously reported (3), we confirmed that levels of I-A^b^ (an MHC class II molecule) on the surface of M1 macrophages were higher than on the surface of M0 or M2 macrophages (**Figure 1A**) and LPS/IL-4-induced macrophages also showed higher levels of I-A^b^ expression than did M0 or M2 macrophages in both C57BL/6 (**Figure 1A**) and Balb/c mice (**Figure S2A**). In contrast, although M1 macrophages showed increased levels of iNOS expression, it was not induced in LPS/IL-4-induced macrophages (**Figure 1A**). In addition, M1 macrophages, but no other macrophages including LPS/IL-4-induced macrophages, showed gene expression of *Il12p40* (**Figure 1B**). Regarding gene expression of *Tnfα*, compared with M0 macrophages that showed marginal levels of *Tnfα* expression, its expression was increased in M1 macrophages, decreased in M2 macrophages, and of a similar level in LPS/IL-4-induced macrophages (**Figure 1B**).

**Figure 2:**
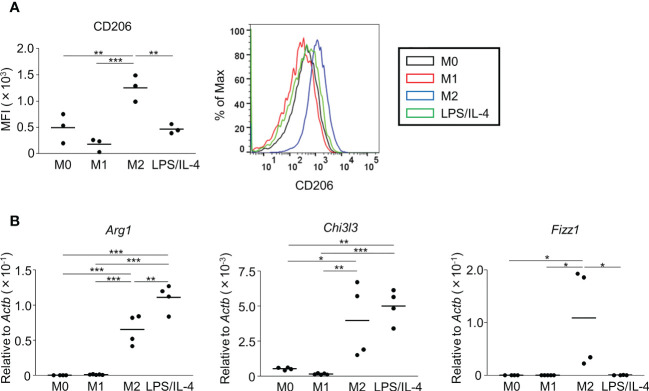
M2 marker expression profiles of M2 macrophages and LPS/IL-4-induced macrophages. Bone marrow-derived macrophages were prepared and stimulated with either LPS, IL-4, or LPS plus IL-4 for 24 h **(A, B)**. **(A)** Flow cytometric analysis was performed to examine the expression of the M2 marker CD206. Mean fluorescence intensity and representative histogram data are shown gated on 7-AAD^−^ CD45^+^ CD11b^+^ F4/80^+^ for CD206. **(B)** Gene expression levels were examined by reverse transcription-quantitative PCR analysis. The expression levels of *Arg1*, *Chi3l3*, and *Fizz1* were normalized to that of *Actb*. Each point represents the data from an individual experiment. Horizontal bars indicate mean value. *P<0.05, **P<0.01, ***P<0.001.

Next, we examined M2 macrophage-related phenotypes. CD206 was expressed at a moderate level on M0 macrophages, and its expression was decreased on M1 macrophages, increased on M2 macrophages, and not significantly different on LPS/IL-4-induced macrophages. Similar results were obtained in C57BL/6 (**Figure 2A**) and BALB/c mice (**Figure S2B**). Regarding gene expressions of M2 markers (*Arg1*, *Chi3l3*, and *Fizz1*), expression of *Arg1* and *Chi3l3* was induced in M2 and LPS/IL-4-induced macrophages, and *Fizz1* was induced in M2 macrophages, but not in LPS/IL-4-induced macrophages (**Figure 2B**).

**Figure 3:**
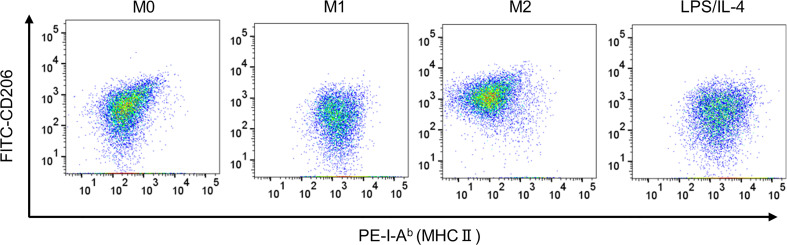
LPS/IL-4-induced macrophages show a homogeneous population. Bone marrow-derived macrophages were prepared and stimulated with either LPS, IL-4, or LPS plus IL-4 for 24 h. Flow cytometric analysis was performed to examine the expression of I-A^b^ and CD206 gated on 7-AAD^−^ CD45^+^ CD11b^+^ F4/80^+^. Representative dot plot data are shown.

We confirmed that LPS/IL-4-induced macrophages were a homogeneous population and not simply a mixture of M1 and M2 phenotypes, at least as determined by the expression of CD206 and I-A^b^ (**Figure 3**). Also, we obtained the same result in BALB/c mice as well as C57BL/6J mice (**Figure S3**). These results suggest that LPS/IL-4-induced macrophages are a subset unique and different from conventional M1 and M2 macrophages.

### 3.2 LPS/IL-4-induced macrophages show high activation levels of glycolysis and oxidative phosphorylation

**Figure 4:**
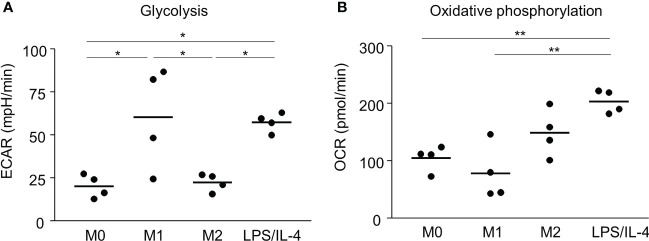
LPS/IL-4-induced macrophages activate both glycolysis and oxidative phosphorylation. Bone marrow-derived macrophages were prepared and stimulated with either LPS, IL-4, or LPS plus IL-4. **(A)** Extracellular acidification rate (ECAR) and **(B)** oxygen consumption rate (OCR) were measured by using a flux analyzer. Results of glycolysis **(A)** and oxidative phosphorylation **(B)** were measured by using an XF Mito Stress Kit. Data are combined from three or four independent experiments. Horizontal bars indicate mean. *P<0.05, **P<0.01.

Previous reports have shown that M1 and M2 macrophages have different metabolic pathways involved in energy production, which is an essential factor controlling macrophage functions (5). For example, glycolysis predominates in M1 macrophages, and oxidative phosphorylation predominates in M2 macrophages (5, 15, 16). Consistent with previous studies, we found that the extracellular acidification rate, which is an indicator of glycolysis, was higher in M1 macrophages than in M0 and M2 macrophages (**Figure 4A**). LPS/IL-4-induced macrophages showed the same results as M1 macrophages (**Figure 4A**). We found that the oxygen consumption rate, which is an indicator of oxidative phosphorylation, tended to be higher in M2 macrophages than in M0 and M1 macrophages, and LPS/IL-4-induced macrophages were significantly higher than that in M0 and M1 macrophages. In addition, oxygen consumption rate in LPS/IL-4-induced macrophages tend to be higher than that in M2 macrophages (**Figure 4B**). Thus, LPS/IL-4-induced macrophages showed a unique pattern of energy metabolism with high activities of both glycolysis and oxidative phosphorylation.

### 3.3 LPS/IL-4-induced macrophages show strong, glycolysis-dependent phagocytic activity

**Figure 5:**
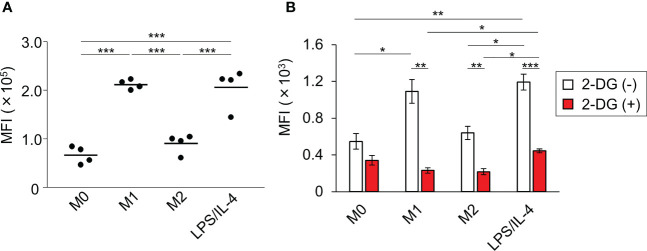
LPS/IL-4-induced macrophages show strong, glycolysis-dependent phagocytic activity. Bone marrow-derived macrophages were prepared and stimulated with either LPS, IL-4, or LPS plus IL-4. **(A)** Phagocytotic assay with fluorescently labeled microbeads (1 µm) was performed. Mean fluorescence intensity is shown on the basis of flow cytometric analysis. Data are combined from two independent experiments. Horizontal bars indicate mean value. ***P<0.001. **(B)** Phagocytotic assay with FITC-staining *E. coli* was performed in the absence or presence of 2-deoxy-D-glucose (2-DG). Results are shown on the basis of flow cytometric analysis. Data are combined from two independent experiments. Horizontal bars indicate mean ± standard error. *P<0.05, **P<0.01, ***P<0.001.

We performed a flow cytometric analysis using fluorescently labeled microbeads to investigate macrophage phagocytotic activity. We found that both M0 and M2 macrophages showed low levels of phagocytosis activity, whereas both M1 and LPS/IL-4-induced macrophages showed activated phagocytosis (**Figure 5A**).

Since the phagocytosis activity of M1 macrophages is known to require glycolysis (5), we next investigated the importance of glycolysis in enhancing the phagocytotic activity observed in LPS/IL-4-induced macrophages. As we found with fluorescently labeled microbeads (**Figure 5A**), large amounts of FITC-staining *E. coli* were taken up by M1 and LPS/IL-4-induced macrophages, and their phagocytotic activity was suppressed by treatment with 2-deoxy-D-glucose (an analog of glucose to inhibit glycolysis) to the same level as M0 macrophages (**Figure 5B**). These findings indicated that, like M1 macrophages, glycolysis was required for the enhanced phagocytotic activity of LPS/IL-4-induced macrophages.

## 4 Discussion

In this study, we showed that when macrophages were stimulated simultaneously with LPS and IL-4, they acquired unique characteristics distinct from those of conventional M1 and M2 macrophages (**Table 1**).

**Table 1:** Summary of LPS/IL-4-induced macrophage features.

			M0	M1 (LPS)	M2 (IL-4)	LPS +IL-4
Cell surface	M1 marker	I-A^b^ (MHC class Il)	Positive	↑↑	(-)	↑↑↑
		iNOS	Negative	↑↑↑	(-)	↑
	M2 marker	CD206	Positive	↓	↑↑	(-)
Gene expression	M1 marker	*Il12-p40*	Negative	↑↑↑	(-)	(-)
		*Tnfα*	Positive	↑↑↑	↓	(-)
	M2 marker	*Arg1*	Negative	(-)	↑↑	↑↑↑
		*Chi3l3*	Positive	↓	↑↑↑	↑↑↑
		*Fizz1*	Negative	(-)	↑	(-)
Phagocytosis (FITC-labeled beads and FITC-staining *Escherichia coli*)			Positive	↑↑↑	(-)	↑↑↑
Energy metabolism		Glycolysis	Positive	↑↑↑	(-)	↑↑↑
		Oxidative phosphorylation	Positive	↓	↑↑	↑↑↑

(-), No change compared to M0; ↑, Increase; ↓, Decrease. Number of arrows shows degree of “Increase” and “Decrease”.

As mechanisms underlying inflammatory cytokine production (e.g., *Tnfα* and *Il12p40)*, it was known that LPS induces proinflammatory cytokines *via* NF-κB pathway (17–19). On the other hands, IL-4 activates STAT6 to interact with NF-κB pathway (20). As previously indicated, STAT6 phosphorylation was increased in LPS/IL-4-induced macrophages as M2 macrophages; however, the level of NF-κB p65 phosphorylation was not significantly changed in LPS/IL-4-induced macrophages in comparison to M1 macrophages (**Figure S4**). In this issue, previous studies showed that not only NF-κB pathway but also LPS-induced STAT1 activation also involves in induction of *Tnfα* and *Il12p40* (21, 22) and that IL-4-induced STAT6 also suppressed STAT1-dependent transcription (23). We found the decreased level of STAT1 phosphorylation in LPS/IL-4-induced macrophages in comparison to M1 macrophages (**Figure S4**). Taken together, it is likely that phosphorylated STAT6 play an important role in regulating the levels of *Tnfα* and *Il12p40* by inhibiting STAT1 phosphorylation in LPS/IL-4-induced macrophages.

Regarding *Arg1*, we examined expression level of *SOCS1*, a critical factor for the expression of *Arg1* (24). As expected, the expression level of SOCS1 was induced in both M2 and LPS/IL-4-induced macrophages (**Figure S5**). We also found that gene expression level of STAT3, which was previously reported to promote the Arg1 expression (25), was specifically higher in LPS/IL-4-induced macrophages than any other macrophages (**Figure S5**). These results suggest that not only SOCS1 but also STAT3 signaling is involved in promoting Arg1 expression in LPS/IL-4-induced macrophages.

Various bacterial infection models using iNOS-deficient mice have shown that iNOS is an important enzyme regulating production of NO for protection against pathogenic microorganisms (26, 27). However, iNOS is not the only critical factor; Arg-1 is also critical for regulation of NO production because IL-4-stimulated bone marrow-derived macrophages (BMDM) from Arg1^flox/flox^ mice can produce NO whereas BMDM from Arg1^+/+^ mice cannot (28). Our findings of very low expression of iNOS in comparison to M1 macrophages and very increased levels of *Arg1* in LPS/IL-4-induced macrophages in comparison to M2 macrophages suggest that LPS/IL-4-induced macrophages barely produce NO. In addition, L-arginine metabolism in LPS/IL-4-induced macrophages might tend to produce arginine metabolites such as urea or ornithine rather than NO. A number of studies have shown a close relationship between an increase in arginine metabolism and fibrogenesis. For example, arginine metabolites, such as ornithine and citrulline, are induced in peripheral blood in the bleomycin-induced pulmonary fibrosis murine model (29). Another study showed that levels of arginine metabolites were higher in human patients with idiopathic pulmonary fibrosis than in normal subjects (30). In addition, in pulmonary fibrosis, iNOS and Arg1 are regarded as being in a competitive relationship for arginine, and Arg1 is upregulated in M2 macrophages localized in fibrotic lesions (31, 32). In this study, LPS/IL-4-induced macrophages showed upregulation of *Arg1* compared to M2 macrophages, despite expression of iNOS being very low, suggesting that both M2 and LPS/IL-4-induced macrophages may be involved in the pathogenesis of pulmonary fibrosis.

Our results showed that LPS/IL-4-induced macrophages exhibited a unique energy metabolism unlike that of M1 or M2 macrophages. Recently, it has become clear that the type of energy metabolism on which macrophages are dependent, such as glycolysis, the TCA cycle, or oxidative phosphorylation, varies depending on the activation state and subset of the macrophages (5). For example, it has become clear that M1 macrophages’ dependency on glycolysis is due to their selective overexpression of the active isoform of phosphofructokinase, which is the rate-limiting step of glycolysis, and to their inhibition of aconitase 2 and pyruvate dehydrogenase with large amounts of NO (33, 34). M2 macrophages have high levels of CD36 expression on the surface for taking up triglycerides, which activate oxidative phosphorylation by promoting β-oxidation (35). With respect to the correlation between energy metabolism and function, M1 macrophages promote glycolysis to enhance NADPH production *via* activation of the pentose phosphate pathway and increase ROS production *via* NOX (5). M2 macrophages enhance oxidative phosphorylation to participate in inflammatory convergence and tissue repair (5, 6). On the other hand, LPS/IL-4-induced macrophages exhibit a unique type of energy metabolism that strongly activates both glycolysis and oxidative phosphorylation, which is very different from that of M1 and M2 macrophages. The energy metabolism of LPS/IL-4-induced macrophages may influence their phenotype and their function.

In terms of phenotype, M2 macrophages show a STAT6-dependent increase in the level of CD206 expression (36); however, the level of CD206 expression in LPS/IL-4-induced macrophages were suppressed to a level equivalent to that of M0 macrophages. Also, expression of *Fizz1* was not observed in LPS/IL-4-induced macrophages even though it was upregulated in M2 macrophages. These results suggest that glutamine metabolism, which is known to be active in M2 macrophages, may be deeply involved. It is reported that glycosylation with uridine diphosphate-N-acetylglucosamine (UDP-GlcNAc) is required to express CD206, and inhibition of the glycosylation suppresses not only CD206 but also protein expression of FIZZ1 (37). In addition, α-ketoglutarate, which is one of the main substrates for the TCA cycle, is synthesized from glutamine as a substrate (37). Therefore, M2 macrophages supply glutamine for UDP-GlcNAc synthesis to promote expression of CD206 and FIZZ1 and for α-ketoglutarate synthesis to enhance oxidative phosphorylation. On the other hand, oxidative phosphorylation in LPS/IL-4-induced macrophages tended to be higher than in M2 macrophages. Furthermore, LPS/IL-4-induced macrophages were not observed to express *Fizz1*. These results suggest that glutamine metabolism in LPS/IL-4-induced macrophages might favor α-ketoglutarate synthesis over UDP-GlcNAc synthesis. However, although this result is considered to be related to the suppression of the synthesis of UDP-GlcNAc, the mechanism of suppression at the level of *Fizz1* gene expression remains to be clarified, and further studies will be needed to verify the underlying mechanisms.

In terms of function, we found that LPS/IL-4-induced macrophages exhibited strong phagocytotic activity, as did M1 macrophages, and that the glycolytic pathway is required for the enhancement of this activity. The importance of the glycolytic pathway in phagocytosis has previously been demonstrated (15) and our results are consistent with the results of that study. In general, pathogens ingested by macrophages is broken down by NO and ROS (5). From our results, it is highly probable that the NO production capacity of LPS/IL-4-induced macrophages is lower than that of M1 macrophages because of decreased iNOS expression. On the other hand, since the activation level of the glycolytic pathway was similar to that of M1 macrophages, it is expected that the NOX-mediated ROS production capacity through activation of the pentose phosphate cycle may be similar to that of M1 macrophages. Furthermore, it is known that the ROS that kills pathogens is produced not only *via* NOX-dependent pathways but also *via* mitochondria (so-called mitochondrial ROS, mROS) (38). The activation level of oxidative phosphorylation was higher in LPS/IL-4-induced macrophages than in the other macrophages, suggesting that mROS production might be increased. Therefore, although LPS/IL-4-induced macrophages might have a lower NO production capacity than M1 macrophages, the total production of ROS, including mROS, is expected to be higher than that of M1 macrophages. In the future, we would like to study the bactericidal ability after phagocytosis by LPS/IL-4-induced macrophages, in relation not only to NO production but also to ROS and mROS production.

Some types of macrophages are known to exhibit different phenotypes in comparison to M1 and M2 macrophages (39). For example, macrophages isolated from the resolving phase of acute inflammation showed both high levels of iNOS and anti-inflammatory IL-10, M1 and M2 marker, respectively (40). Another study showed that macrophages in the skin from patients with a chronic venous leg ulcers also expressed both M1 marker (TNF-α and IL-12p40) and M2 marker (arginase and CD206) (41). Moreover, CX3CR1^hi^ Ly6C^int^ F4/80^+^ I-A^+^/I-E^+^ macrophages were discovered as a new osteoclast precursor, and disease-specific macrophages involved in the pathogenesis of allergy, fibrosis, and metabolic syndrome were also found despite being categorized as a conventional M2 macrophages (42–46). Similar to our experimental condition, it can be speculated that unique macrophages were induced by LPS and IL-4, in the lung with the asthma with bacterial infection (9, 47).

In this study, in comparison to M2 macrophages, LPS/IL-4-induced macrophages showed increased expression of *Arg1* whose protein involves in deposition of collagenous and extracellular matrix components in lung parenchyma to make asthma severe (48, 49). In addition, LPS/IL-4-induced macrophages showed no expression of *Fizz1* whose protein involves in limiting the pathogenesis of Th2 cytokine-mediated pulmonary inflammation (50). Furthermore, the energy metabolism of LPS/IL-4-induced macrophages was different from M1 and M2 macrophages, suggesting that they are a unique subset in comparison to M1 and M2 macrophages. In the future, we will try to elucidate the functional roles of LPS/IL-4-induced macrophages *in vivo*.

In conclusion, our study showed that macrophages induced by simultaneous stimulation with LPS and IL-4 were different from M1 and M2 macrophages in the phenotype markers and energy metabolism and showed strong phagocytosis activity. We hope that our study can be a part of new finding for understanding the diversity of macrophages.

## Data availability statement

The original contributions presented in the study are included in the article/**Supplementary Material**. Further inquiries can be directed to the corresponding author.

## Ethics statement

The animal study was reviewed and approved by The Animal Care and Use Committee of the National Institutes of Biomedical Innovation, Health, and Nutrition Approval No. DSR04-37R1 and were conducted in accordance with their guidelines.

## Author contributions

KI, TN, and JK conceived and designed the study, performed the data analysis, and wrote the manuscript. KI, TN, AS, SK, EN, and KH performed the experiments and discussed the results. All authors contributed to the article and approved the submitted version.

## Supplementary material

Supplementary Figure 1 Morphology of prepared macrophage. Bone marrow-derived macrophages were prepared and stimulated with either LPS, IL-4, or LPS plus IL-4. Data are representative of 3 independent experiments. Scale bars 50 μm.

Supplementary Figure 2 Representative M1 and M2 marker expression in LPS/IL-4-induced macrophages derived from BALB/c mice. Bone marrow-derived macrophages derived from BALB/c mice were prepared and stimulated with either LPS, IL-4, or LPS plus IL-4 for 24 h. Flow cytometric analysis was performed to examine the expression of M1 marker (i.e., I-A^b^) **(A)** and M2 markers (i.e., CD206) **(B)**. Mean fluorescence intensity (MFI) and representative histogram data are shown and gated on 7-AAD^−^ CD45^+^ CD11b^+^ F4/80^+^ for I-A^b^ and CD206. Data are combined from two independent experiments. Horizontal bars indicate mean value. **P<0.05, **P<0.01*, *** *P*<0.001.

Supplementary Figure 3 LPS/IL-4-induced macrophages derived from BALB/c mice show a homogeneous population. Bone marrow-derived macrophages derived from BALB/c mice were prepared and stimulated with either LPS, IL-4, or LPS plus IL-4 for 24 h. Flow cytometric analysis was performed to examine the expression of I-A^b^ and CD206 gated on 7-AAD^−^ CD45^+^ CD11b^+^ F4/80^+^. Representative dot plot data are shown.

Supplementary Figure 4 LPS/IL-4-induced macrophages show increased of STAT6 phosphorylation with decreased expression of STAT1 phosphorylation but not p65 phosphorylation. Each cell was stimulated with either LPS, IL-4, or LPS plus IL-4 for 1 h to assess p65, phosphorylated p65 (p-p65), STAT6, phosphorylated STAT6 (p-STAT6) and β-actin, and for 3 h to assess STAT1, phosphorylated STAT1 (p-STAT1) and β-actin. The cell lysates were analyzed by a capillary-based immunoassay with each antibody. Electropherograms could be visualized by calculating chemiluminescence intensity on the Compass Simple Western software. Representative data from 3 independent experiments are shown. Ratio to phosphorylation: non-phosphorylation was calculated by chemiluminescence intensity of each protein. Data were combined from three independent experiments. Data indicate mean ± standard error. * *P*<0.05, ** *P*<0.01, *** *P*<0.001.

Supplementary Figure 5 The expression levels of *SOCS1* and *STAT3* in LPS/IL-4-induced macrophages. Bone marrow-derived macrophages derived from C57BL/6J mice were prepared and stimulated with either LPS, IL-4, or LPS plus IL-4 for 24 h. Gene expression levels of *SOCS1* and *STAT3* were examined by reverse transcription-quantitative PCR analysis. The genes were normalized to that of *Actb*. Each point represents the data from an individual experiment. Horizontal bars indicate mean value. * *P*<0.05, ** *P*<0.01.
