# Supplementary figures and images for: Induction of unique macrophage subset by simultaneous stimulation with LPS and IL-4
Source: Front Immunol. 2023 Apr 21;14:1111729. doi: 10.3389/fimmu.2023.1111729 (PMC10167635; doi:10.3389/fimmu.2023.1111729)

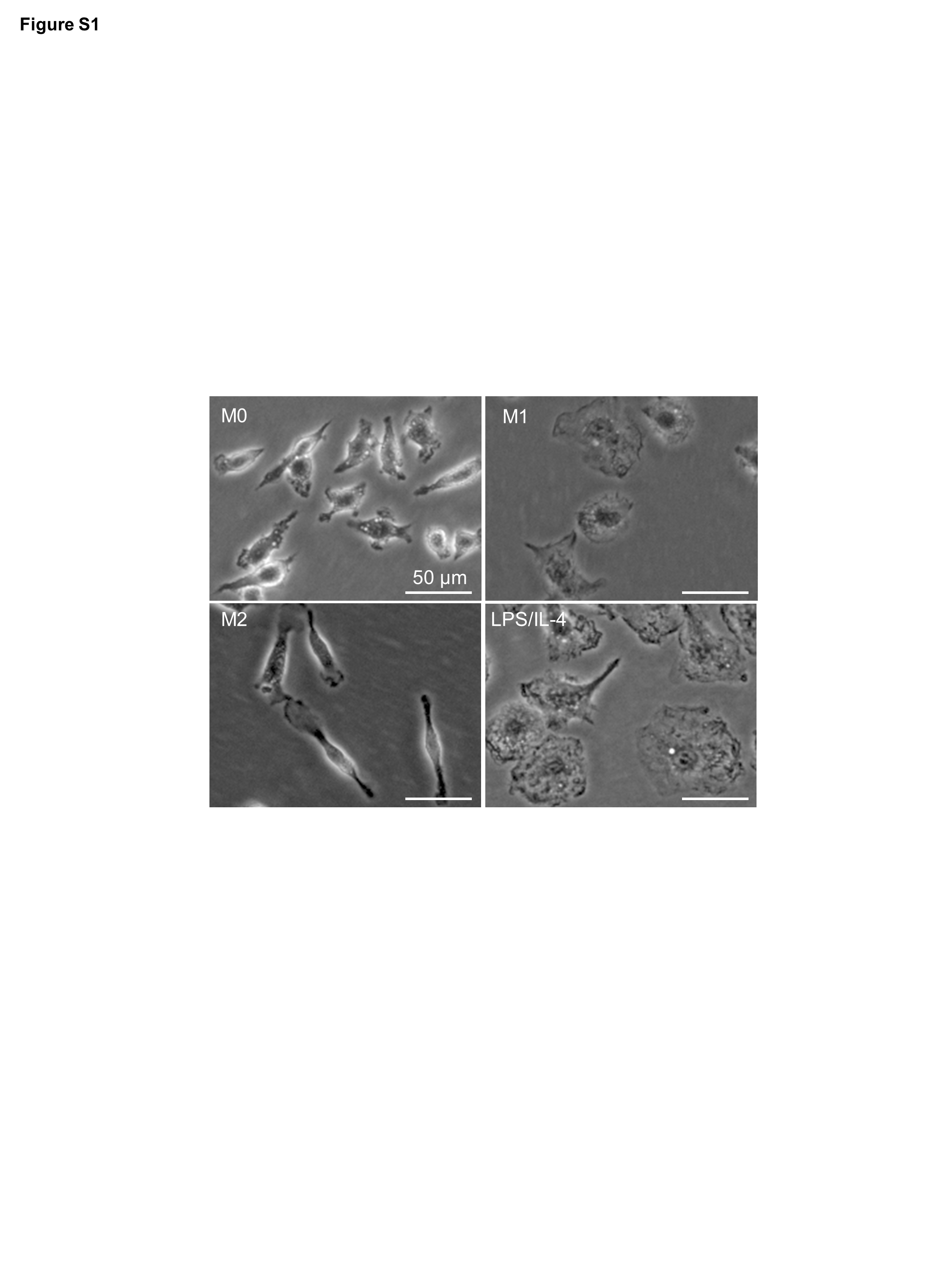

Supplement: Supplementary Figure 1 — Morphology of prepared macrophage. Bone marrow-derived macrophages were prepared and stimulated with either LPS, IL-4, or LPS plus IL-4. Data are representative of 3 independent experiments. Scale bars 50 μm. [file Image_1.tif]

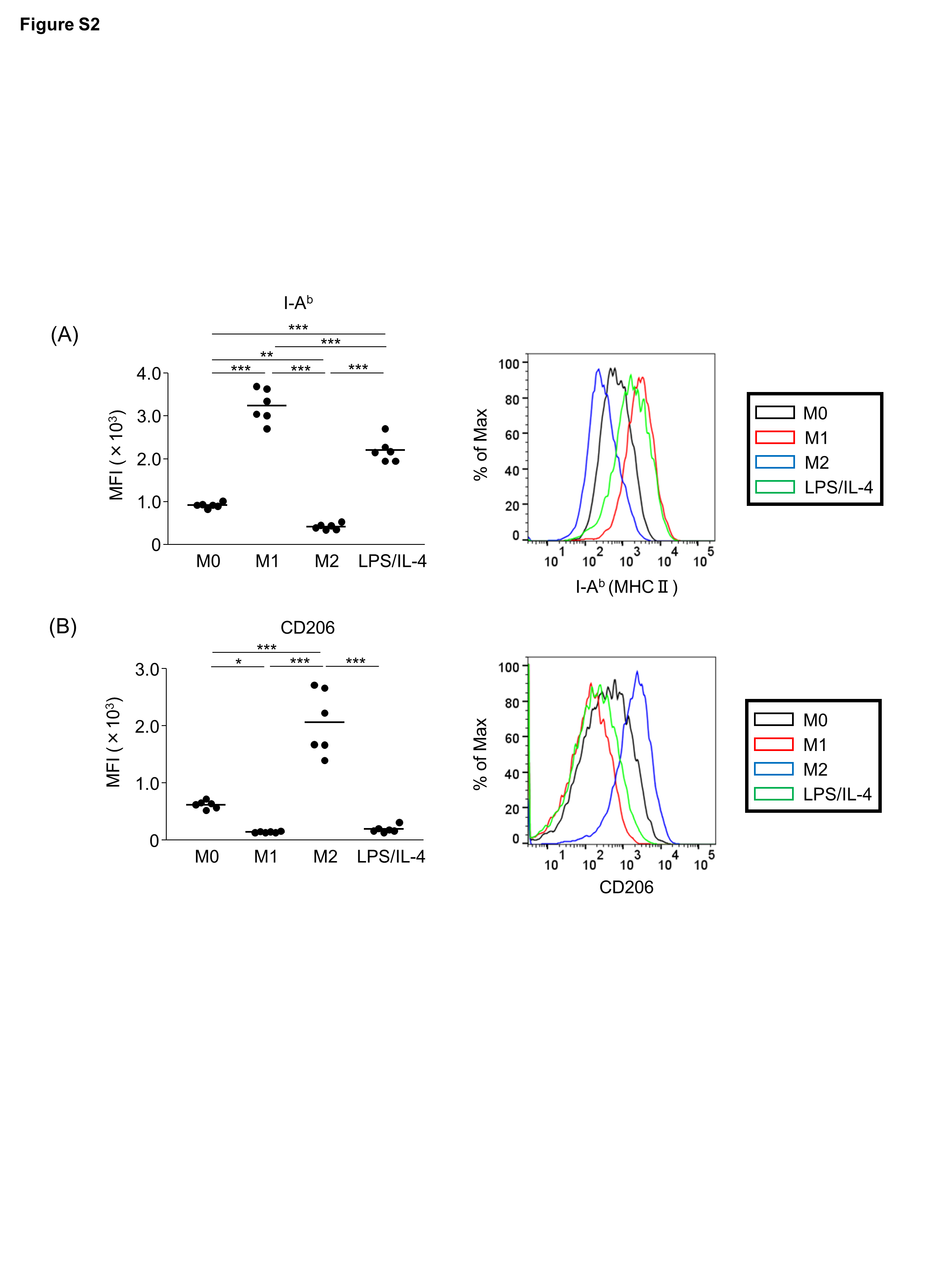

Supplement: Supplementary Figure 2 — Representative M1 and M2 marker expression in LPS/IL-4-induced macrophages derived from BALB/c mice. Bone marrow-derived macrophages derived from BALB/c mice were prepared and stimulated with either LPS, IL-4, or LPS plus IL-4 for 24 h. Flow cytometric analysis was performed to examine the expression of M1 marker (i.e., I-Ab) (A) and M2 markers (i.e., CD206) (B). Mean fluorescence intensity (MFI) and representative histogram data are shown and gated on 7-AAD− CD45+ CD11b+ F4/80+ for I-Ab and CD206. Data are combined from two independent experiments. Horizontal bars indicate mean value. *P<0.05, **P<0.01, *** P<0.001. [file Image_2.tif]

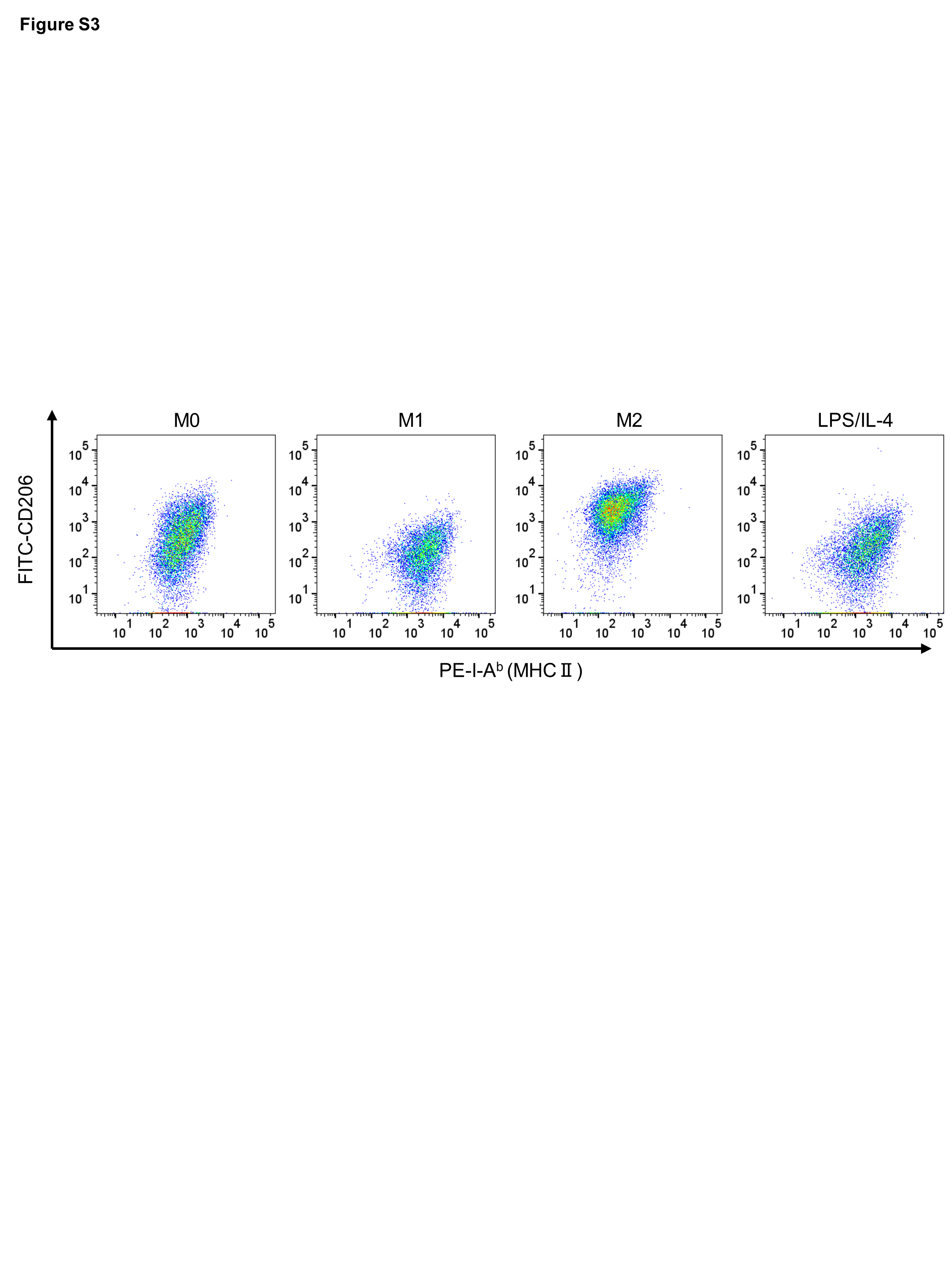

Supplement: Supplementary Figure 3 — LPS/IL-4-induced macrophages derived from BALB/c mice show a homogeneous population. Bone marrow-derived macrophages derived from BALB/c mice were prepared and stimulated with either LPS, IL-4, or LPS plus IL-4 for 24 h. Flow cytometric analysis was performed to examine the expression of I-Ab and CD206 gated on 7-AAD− CD45+ CD11b+ F4/80+. Representative dot plot data are shown. [file Image_3.tif]

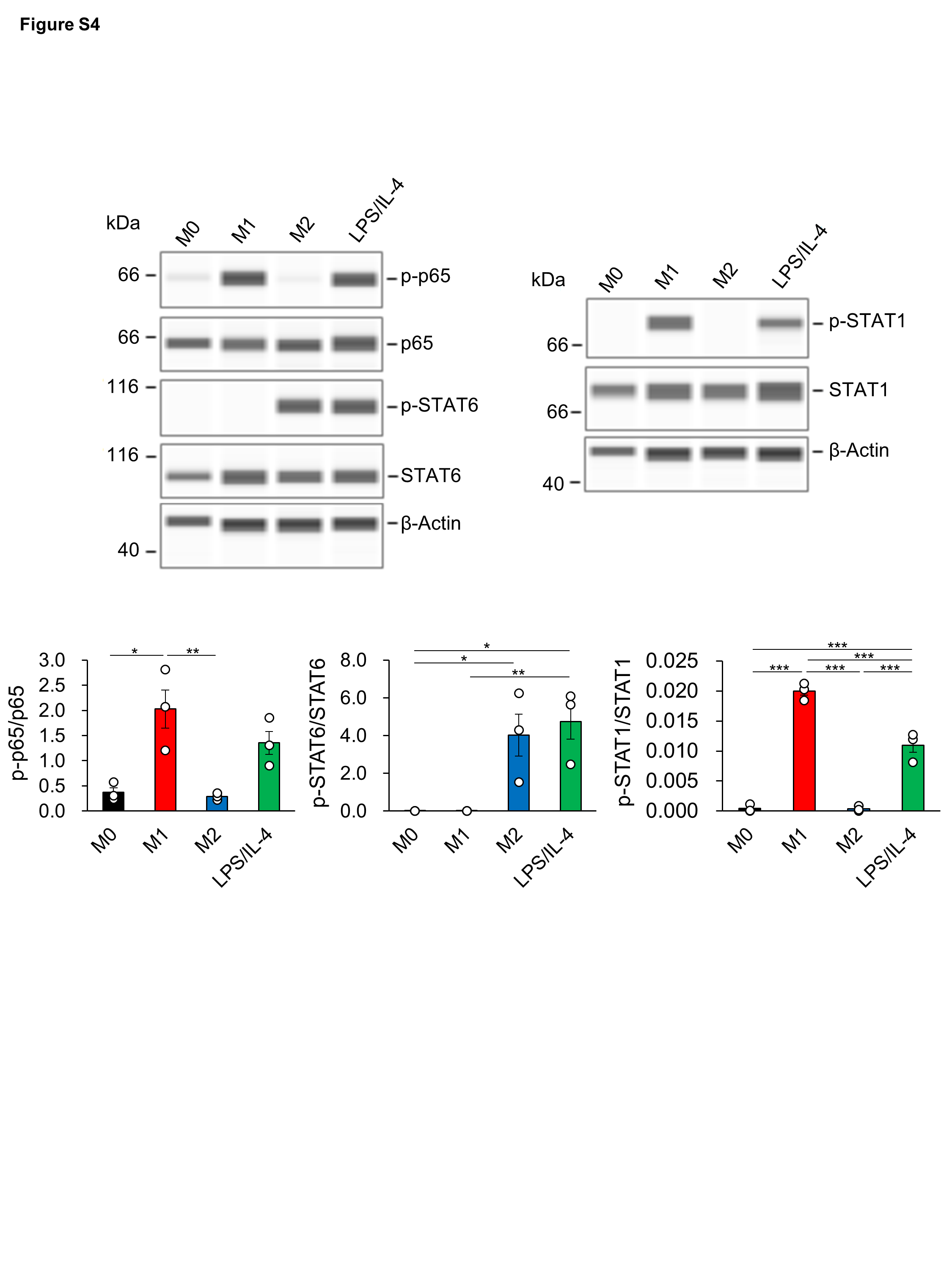

Supplement: Supplementary Figure 4 — LPS/IL-4-induced macrophages show increased of STAT6 phosphorylation with decreased expression of STAT1 phosphorylation but not p65 phosphorylation. Each cell was stimulated with either LPS, IL-4, or LPS plus IL-4 for 1 h to assess p65, phosphorylated p65 (p-p65), STAT6, phosphorylated STAT6 (p-STAT6) and β-actin, and for 3 h to assess STAT1, phosphorylated STAT1 (p-STAT1) and β-actin. The cell lysates were analyzed by a capillary-based immunoassay with each antibody. Electropherograms could be visualized by calculating chemiluminescence intensity on the Compass Simple Western software. Representative data from 3 independent experiments are shown. Ratio to phosphorylation: non-phosphorylation was calculated by chemiluminescence intensity of each protein. Data were combined from three independent experiments. Data indicate mean ± standard error. * P<0.05, ** P<0.01, *** P<0.001. [file Image_4.tif]

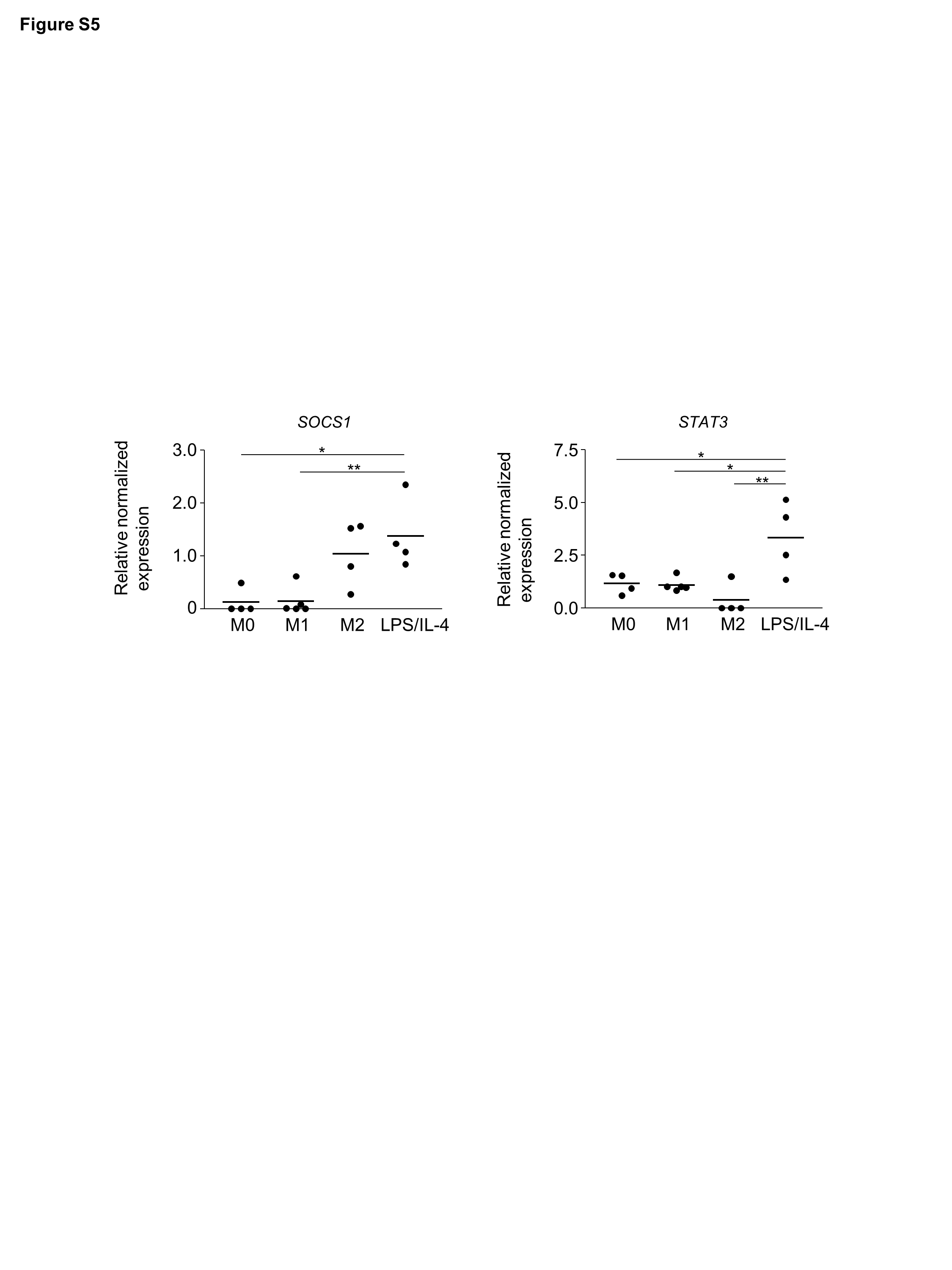

Supplement: Supplementary Figure 5 — The expression levels of SOCS1 and STAT3 in LPS/IL-4-induced macrophages. Bone marrow-derived macrophages derived from C57BL/6J mice were prepared and stimulated with either LPS, IL-4, or LPS plus IL-4 for 24 h. Gene expression levels of SOCS1 and STAT3 were examined by reverse transcription-quantitative PCR analysis. The genes were normalized to that of Actb. Each point represents the data from an individual experiment. Horizontal bars indicate mean value. * P<0.05, ** P<0.01. [file Image_5.tif]
